# Digital Biomarkers for Precision Early Detection of Lung Cancer: Integrating AI‐Driven Multi‐Omics Into Clinical Pathways

**DOI:** 10.1002/cam4.71578

**Published:** 2026-02-05

**Authors:** Fan Bu, Zhi‐Qiang Ling

**Affiliations:** ^1^ Zhejiang Cancer Institute, Zhejiang Cancer Hospital Hangzhou Zhejiang China; ^2^ The Second Clinical Medical College of Zhejiang Chinese Medicine University Hangzhou People's Republic of China

**Keywords:** artificial intelligence, early detection, liquid biopsy, lung cancer, multi‐omics, translational biomarkers

## Abstract

**Background:**

Lung cancer remains the leading cause of cancer‐related mortality worldwide, highlighting the urgent need for earlier detection within real‐world screening and patient management pathways. Recent advances in multi‐omics technologies have created new opportunities for identifying biomarkers associated with early‐stage lung cancer, particularly in high‐risk populations under clinical surveillance.

**Methods:**

This review systematically evaluates early diagnostic biomarkers across multiple omics layers, including genomics, epigenomics, transcriptomics, proteomics, metabolomics and microbiomics. It also summarises the application of artificial intelligence (AI), particularly machine learning and deep learning approaches, for integrating and analysing complex multi‐omics datasets to support biomarker discovery and clinical decision‐making.

**Results:**

Multi‐omics strategies are accelerating the identification of molecular signatures relevant to early lung cancer detection. AI‐driven methods enable the extraction of latent patterns from high‐dimensional data, facilitating risk stratification, diagnostic refinement, histological subtyping and treatment planning. The review highlights the clinical utility of these biomarkers and their potential incorporation into screening algorithms, as well as the development of AI‐based clinical decision support systems (CDSS) aligned with real‐world clinical workflows. However, major barriers to clinical translation remain, including multi‐centre data heterogeneity, limited model interpretability affecting clinical trust, regulatory and cost‐effectiveness challenges and insufficient validation in prospective cohorts.

**Conclusions:**

Emerging technologies, such as single‐cell and spatial multi‐omics, along with federated learning frameworks, offer promising solutions to bridge the gap between computational discovery and clinical implementation. The integration of AI and multi‐omics approaches has the potential to advance risk‐adapted and personalised early detection strategies for lung cancer.

**Abbreviations**
A1AT: alpha‐1‐antitrypsin
AUC: area under the curve
BAL: bronchoalveolar lavage
CA125: cancer antigen 125
CDSS: clinical decision support system
CE: cholesteryl ester
CEA: carcinoembryonic antigen
cfRNA: cell‐free RNA
CgA: chromogranin A
CGI: CpG Island
CIS: carcinoma in situ
CNV: copy number variation
CT: computed tomography
ctDNA: circulating tumour DNA
CTSD: cathepsin D
CYFRA 21‐1: cytokeratin‐19 fragment
ddPCR: Droplet Digital Polymerase Chain Reaction
DL: deep learning
DNMT: DNA methyltransferase
DNN: deep neural network
EGFR: epidermal growth factor receptor
EHR: electronic health record
FTICR‐MS: Fourier Transform Ion Cyclotron Resonance Mass Spectrometry
GAS5: growth arrest‐specific 5
GlcNAc: N‐acetylglucosamine
H&E: haematoxylin and eosin
HE4: human epididymis protein 4
HER: human epidermal growth factor receptor
HSP60: heat shock protein 60
IRB: Institutional Review Board
KL Divergence: Kullback–Leibler divergence
LC–MS: liquid chromatography–mass spectrometry
LDCT: low‐dose computed tomography
lncRNA: long non‐coding RNA
LPC: lysophosphatidylcholine
LPE: lysophosphatidylethanolamine
LUAD: lung adenocarcinoma
LUSC: lung squamous cell carcinoma
miRNA: microRNA
ML: machine learning
MRM: multiple reaction monitoring
MSI: microsatellite instability
MSP: methylation‐specific PCR
ncRNA: non‐coding RNA
NGS: next‐generation sequencing
NSCLC: non‐small cell lung cancer
NSE: neuron‐specific enolase
OMICS: comprehensive molecular profiling (genomics, transcriptomics, etc.)
OTU: operational taxonomic unit
PC: phosphatidylcholine
PE: phosphatidylethanolamine
PET‐CT: positron emission tomography–computed tomography
PON1: paraoxonase 1
PPV: positive predictive value
ProGRP: Pro‐gastrin‐releasing peptide
qRT‐PCR: quantitative reverse transcription polymerase chain reaction
RB1: retinoblastoma 1
RBP: retinol‐binding protein
ROC: receiver operating characteristic
scATAC‐seq: single‐cell assay for transposase‐accessible chromatin sequencing
SCCAg: squamous cell carcinoma antigen
SCLC: small cell lung cancer
scRNA‐seq: single‐cell RNA sequencing
SM: sphingomyelin
TCGA: The Cancer Genome Atlas
TP53: tumour protein 53
VOC: volatile organic compound
WES: whole‐exome sequencing
XAI: explainable artificial intelligence

## 1 Introduction

Lung cancer remains the leading cause of cancer‐related mortality globally, driven by a critical lack of robust early detection tools and profound tumour heterogeneity [1]. Although low‐dose computed tomography (LDCT) screening reduces mortality, its clinical implementation faces substantial limitations—including high false‐positive rates and overdiagnosis—restricting widespread adoption [2]. Clinically viable, non‐invasive diagnostics with improved accuracy are urgently needed to detect early‐stage disease when interventions are most effective.

High‐throughput multi‐omics approaches (genomics, epigenomics, transcriptomics, proteomics, metabolomics, microbiomics) now enable comprehensive molecular profiling of lung cancer, revealing novel biomarkers linked to disease mechanisms and progression [3, 4]. However, integrating these complex, high‐dimensional datasets requires advanced computational frameworks. Artificial intelligence (AI), particularly machine learning (ML) and deep learning (DL), provides transformative solutions by identifying latent biological patterns and developing high‐accuracy diagnostic models from multi‐omics data [5, 6]. Critically, AI‐powered liquid biopsy analysis—interrogating circulating tumour DNA (ctDNA), microRNAs and extracellular vesicles—offers minimally invasive, real‐time detection with high translational potential [7].

Multi‐omics integration refers to the systematic combination of heterogeneous biological data layers—such as genomics, epigenomics, transcriptomics, proteomics and metabolomics—within a unified analytical framework to capture complementary disease‐relevant signals. While several recent reviews have independently summarised advances in AI algorithms or individual omics‐based biomarkers for lung cancer, few have systematically examined how AI‐driven multi‐omics integration can be translated into real‐world early detection and clinical screening pathways. Moreover, the clinical challenges of model interpretability, data heterogeneity and regulatory validation are often underrepresented in prior reviews. In this context, our review uniquely emphasises the clinical applicability, translational bottlenecks and decision‐support relevance of AI‐enabled multi‐omics biomarkers for early lung cancer detection.

**FIGURE 1:**
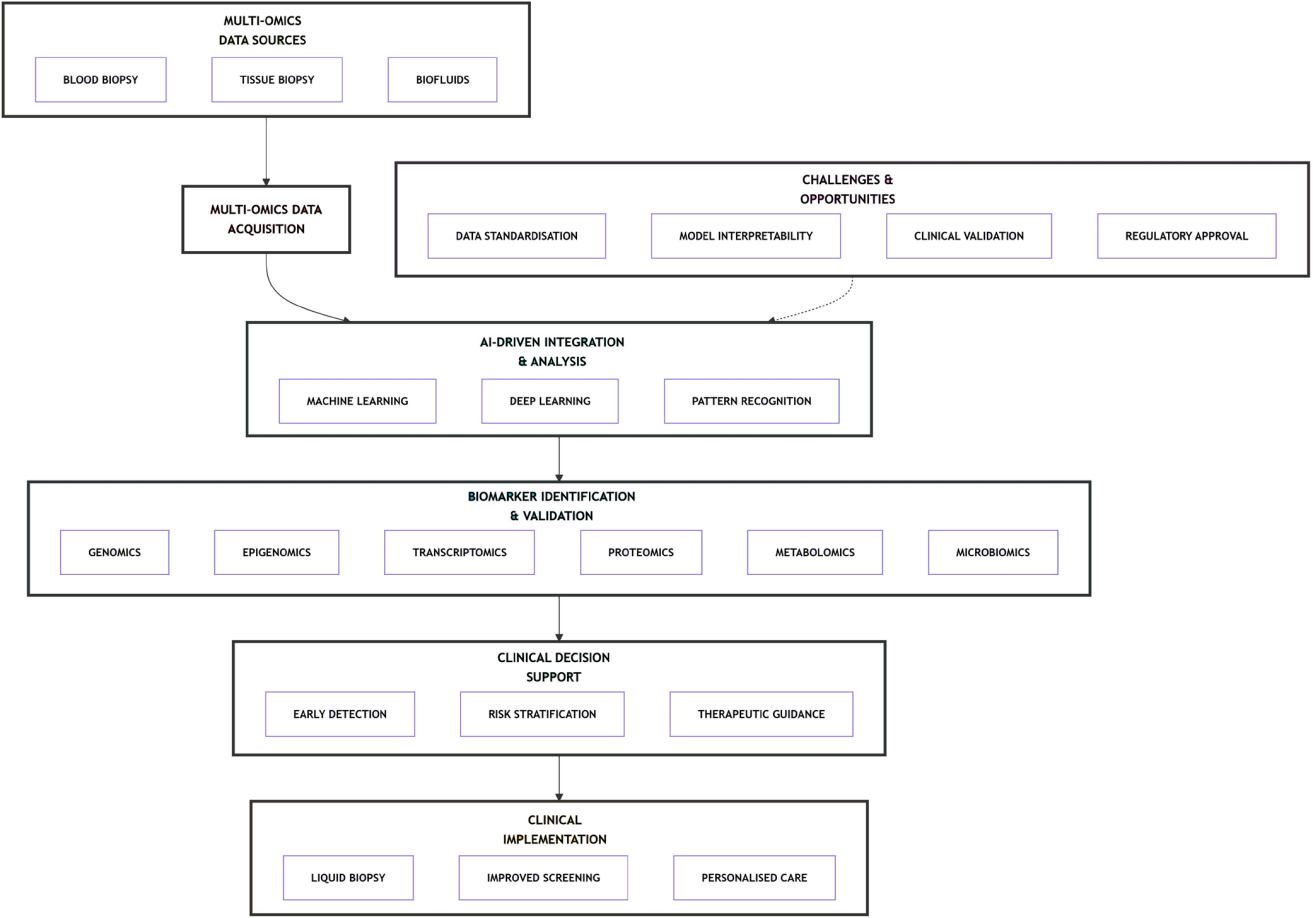
The structural flow of this review, outlining the analytical framework from multi‐omics biomarker discovery to clinical AI integration.

This review synthesises advances in AI‐driven multi‐omics integration for early lung cancer detection. We critically evaluate diagnostic biomarkers across omics layers, assess AI's role in biomarker discovery and clinical decision support, and discuss translational challenges and emerging opportunities. Figure 1 outlines our analytical framework: from multi‐omics biomarker discovery to clinical AI integration.

## 2 Multi‐Omics Biomarkers for Early Lung Cancer Detection

### 2.1 Genomics and Epigenomics

#### 2.1.1 Genomic Biomarkers

Cigarette smoking is the principal etiological factor for lung cancer, driving tobacco smoke‐induced genomic and epigenetic alterations that fundamentally modify cellular genetic programs [1]. Lung cancer develops through a stepwise molecular pathogenesis characterised by the sequential accumulation of genetic and epigenetic alterations driving malignant transformation [8, 9]. This histological continuum—spanning bronchial epithelial hyperplasia, metaplasia, dysplasia, carcinoma in situ (CIS) and invasive carcinoma—is defined by stage‐specific molecular biomarkers, including driver gene mutations (e.g., *EGFR*, *TP53*), DNA methylation changes and microsatellite instability.

Recurrent alterations in proto‐oncogenes—particularly *MYC* amplification, *RAS* family mutations (*KRAS*/*NRAS*) and *HER* family dysregulation—drive oncogenic signalling in lung cancer. Complementary tumour suppressor inactivation, most frequently affecting *TP53*, *RB1* and *CDKN2A* (*p16*), promotes uncontrolled proliferation and genomic instability [10, 11]. Using a multi‐target PCR approach (including *TP53* sequencing, *KRAS* mutation analysis, *p16* methylation‐specific PCR [MSP] and microsatellite instability [MSI] assessment), Ahrendt and colleagues detected tumour‐associated genetic alterations in bronchoalveolar lavage (*BAL*) fluid from patients with non‐small cell lung cancer (NSCLC). Comparative analysis of matched tumour tissue, blood and BAL specimens revealed differential detection rates: *TP53* mutations (56%), *KRAS* mutations (27%), MSI (46%) and *p16* methylation (38%). *TP53* mutations predominated in squamous cell carcinoma, whereas *KRAS* alterations were more frequent in adenocarcinoma.

Several other oncogenic alterations are known critical drivers in lung adenocarcinoma, including mutations in *BRAF*, *ERBB2* and *MET*, as well as *RET* rearrangements. However, few studies have simultaneously investigated multiple oncogenic drivers [12, 13, 14, 15, 16]. Next‐generation sequencing (NGS) technologies provide unprecedented opportunities to delineate the mutational landscape of lung cancer and enhance understanding of genomic alterations relevant to diagnosis. Recent studies have identified a spectrum of genetic alterations, including gene amplifications (*CCND1–3*, *CDK4*, *FGFR1–3*, *MET*, *PDGFRA*, *PIK3CA*, *SOX2*), gene fusions (*FGFR3–TACC3*), tumour suppressor gene mutations (*PTEN*, *TP53*) and point mutations (*EPHA2*, *AKT1*, *DDR2*). Combined assessment of these alterations demonstrates enhanced diagnostic value [17].

Key driver gene alterations central to lung carcinogenesis include mutations in *EGFR*, *KRAS* and *BRAF*; rearrangements or amplifications of *HER2*, *ALK*, *ROS1* and *RET*; and *MET* amplifications or exon 14 skipping mutations. Collectively, these alterations constitute the core genomic landscape of lung cancer [12].

#### 2.1.2 DNA Methylation

DNA methylation, encompassing both hypermethylation and hypomethylation, is a fundamental epigenetic modification critical to the initiation and progression of virtually all human tumours [3]. Epigenetic mechanisms drive tumorigenesis through three principal pathways: promoter hypermethylation, global genomic hypomethylation and histone modifications [3].

Aberrant DNA methylation, catalysed by DNA methyltransferases (DNMTs), predominantly involves hypermethylation of tumour suppressor gene promoters, contributing to the pathogenesis of multiple cancers, including NSCLC [18, 19]. Methylation alterations occur primarily at CpG islands (CGIs) in gene promoter regions. This specificity renders them suitable biomarkers highly amenable to PCR‐based detection, offering significant potential for developing novel methylation‐based biomarkers to enhance early lung cancer diagnosis [20]. In lung cancer, methylation alterations in individual genes or gene panels correlate with therapeutic response and demonstrate clinical utility. Methylation of *RASSF1A*, *SHOX2* and *PTGER4* has been extensively validated as an early diagnostic biomarker panel for this disease; AUC increased from 0.69 to 0.74 [21, 22]. Multigene methylation panels demonstrate enhanced sensitivity and specificity compared to single‐gene assays.

A prospective study analysing promoter methylation of *TAC1*, *HOXA17* and *SOX17* in sputum samples from 150 patients with NSCLC and 60 healthy controls demonstrated 98% sensitivity and 71% specificity [23]. Another study assessing a multigene methylation panel (including *SOX17*, *HOXA9*, *AJAP1*, *PTGDR*, *UNCX* and *MARCH11*) showed 96.7% sensitivity and 60% specificity for NSCLC diagnosis [24]. Liu and colleagues reported that a methylation panel (*PCDHGB6*, *HOXA9*, *MGMT* and miR‐126) achieved 85.2% sensitivity and 81.5% specificity for NSCLC detection [24, 25].

Studies demonstrate elevated *CDKN2A* methylation levels in sputum samples collected up to 3 years before lung cancer diagnosis [26]. A meta‐analysis by Huang and colleagues identified differentially methylated genes across histological subtypes of NSCLC [4]. This analysis revealed two hypomethylated genes (*CDKN2A*, *MGMT*) and three hypermethylated genes (*CDH13*, *RUNX3*, *APC*) in adenocarcinoma versus squamous cell carcinoma. *CDH13* and *APC* demonstrated high sensitivity (0.74 and 0.65) and specificity (0.49 and 0.60), supporting their potential as histology‐specific diagnostic biomarkers for NSCLC (AUC = 0.68 and 0.66) [27].

#### 2.1.3 Histone Modifications

Histone modifications critically regulate lung cancer initiation and progression by remodelling chromatin architecture and fine‐tuning gene expression, thereby modulating tumourigenic processes. These post‐translational alterations dynamically regulate histone‐DNA interactions to govern chromatin accessibility and transcriptional activity. Methylation and acetylation patterns on histone H3 and H4 residues are promising diagnostic biomarkers for lung cancer [28]. Specific aberrations, including elevated H3K27me3 levels, are frequently dysregulated in this disease [29]. *YEATS2* amplification, prevalent in lung cancer, critically regulates tumourigenesis [30].

### 2.2 Transcriptomics and Non‐Coding RNAs

#### 2.2.1 Transcriptomics

Geng and colleagues investigated microRNAs (miRNAs) as diagnostic biomarkers for NSCLC. In a training cohort (*n* = 50), qRT‐PCR analysis of blood samples identified a five‐miRNA panel (*miR‐20a*, *miR‐223*, *miR‐21*, *miR‐221*, *miR‐145*), subsequently validated in a larger cohort. *MiR‐20a*, *miR‐223* and *miR‐145* showed high diagnostic performance, with AUCs of 0.89, 0.94 and 0.92, respectively, indicating strong potential as non‐invasive biomarkers for early NSCLC detection [31].

Lu and colleagues analysed 1132 subjects, identifying six diagnostic miRNA candidates through initial microarray screening of 723 molecules followed by qRT‐PCR validation. Two logistic regression models based on these miRNAs demonstrated that *miR‐17*, *miR‐190b* and *miR‐375* effectively discriminated SCLC from NSCLC, achieving AUCs of 0.878 (training) and 0.869 (validation), highlighting their potential for early detection and histological subtyping [32].

Zhang and colleagues identified 16 candidate miRNAs via microarray screening, validating six by qRT‐PCR. *MiR‐3149* and *miR‐4769‐3p* showed significant upregulation in NSCLC patients and strong discriminative capacity (AUCs 0.830 and 0.735), supporting their utility as early detection biomarkers [33].

Powrozek and colleagues reported significant upregulation of *miR‐944* and *miR‐3662* in lung cancer, with *miR‐944* demonstrating high diagnostic accuracy for squamous cell carcinoma (AUC 0.982) and *miR‐3662* for adenocarcinoma (AUC 0.926) [34]. Similarly, Singh and colleagues analysed six blood‐based miRNAs in adenocarcinoma and squamous cell carcinoma patients, revealing significant upregulation of *miR‐2114* and *miR‐449c* in adenocarcinoma and *miR‐2115* in squamous cell carcinoma [35].

Kumar and colleagues analysed three miRNAs in 161 tissue samples using TaqMan Advanced miRNA assays. *MiR‐197–3p* and *miR‐375‐3p* were significantly upregulated in tumour resection samples, with *miR‐375‐3p* also elevated in biopsies and demonstrating the highest diagnostic performance (AUC 0.749). These miRNAs may assist in distinguishing squamous cell carcinoma from adenocarcinoma [36].

Nadal and colleagues profiled serum miRNAs in 72 NSCLC patients and 22 healthy controls, establishing a four‐miRNA panel with exceptional diagnostic performance (AUC 0.993) as an auxiliary diagnostic tool [37].

Jin and colleagues investigated exosomal miRNA profiles for differentiating stage I NSCLC histological subtypes. RNA sequencing revealed distinct expression patterns between adenocarcinoma and squamous cell carcinoma. A three‐miRNA panel achieved AUCs of 0.899 (NSCLC), 0.936 (adenocarcinoma) and 0.911 (squamous cell carcinoma) [38].

#### 2.2.2 Non‐Coding RNAs as Potential Diagnostic Biomarkers

Non‐coding RNAs (ncRNAs) show dysregulated expression in lung cancer tissues and are detectable in body fluids (e.g., blood, sputum), highlighting their promise for non‐invasive diagnostics. Among ncRNAs, long non‐coding RNAs (lncRNAs) are particularly promising due to their tissue‐specific expression, which may improve diagnostic specificity.

#### 2.2.3 Diagnostic Potential of lncRNAs

Multiple lncRNAs exhibit significant dysregulation (upregulation or downregulation) in lung cancer compared with normal tissues [39], supporting their utility as molecular indicators for early malignancy detection. Notably, *lncRNA HOTAIR* is upregulated in NSCLC; its plasma levels correlate closely with disease progression and metastasis, indicating predictive value for tumour aggressiveness [40]. Similarly, *GAS5* has emerged as a diagnostic biomarker for NSCLC, with circulating levels strongly linked to disease status. ROC curve analysis for early stage of NSCLC with the combination of GAS5, CEA and CA199 showed that the area under the AUC was 0.734 (95% CI, 0.628–0.839; *p* < 0.0005) [41].

#### 2.2.4 circRNAs as Diagnostic Biomarkers

Circular RNAs (circRNAs), characterised by a covalently closed‐loop structure, possess exceptional stability and resistance to exonuclease degradation. This structural integrity enables them to remain intact in biological fluids, making them ideal diagnostic biomarkers [42]. Specific circRNAs are differentially expressed in lung cancer tissues compared with normal controls. For example, *hsa_circ_0077837* and *hsa_circ_0001821* show high diagnostic accuracy for distinguishing NSCLC from normal tissues, as reflected in their AUC values; serum and serum exosomal hsa_circ_0069313 could differ benign lung tumour and NSCLC with AUC values of 0.803 and 0.749, respectively [43]. Detection of circRNAs in plasma via liquid biopsy offers a minimally invasive diagnostic approach [44] and isolation from plasma‐derived exosomes provides an even less invasive alternative to tissue biopsies. Importantly, several circRNAs are upregulated during early‐stage lung adenocarcinoma, underscoring their utility as early diagnostic biomarkers. A recent meta‐analysis reported a pooled diagnostic AUC of 0.78 for circRNAs in Chinese patients with lung cancer, indicating favourable clinical performance [45]. Collectively, these findings suggest circRNAs hold significant promise for enhancing early detection strategies in lung cancer.

### 2.3 Proteomics and Glycomics

#### 2.3.1 Proteomics

Visser and colleagues developed a liquid biopsy‐based decision algorithm for diagnosing lung cancer and differentiating NSCLC from small cell lung cancer (SCLC) [46]. They quantified eight protein tumour markers (*CA125*, *CEA*, *CYFRA 21‐1* and *ProGRP*) using electrochemiluminescence assays and detected *EGFR*, *KRAS* and *BRAF* mutations in plasma circulating tumour DNA (ctDNA) from 1096 patients with suspected lung cancer via droplet digital PCR. Multivariable logistic regression identified *CYFRA 21‐1* as the strongest predictor for NSCLC (AUC = 0.78), whereas *ProGRP* showed the highest sensitivity for SCLC diagnosis (sensitivity 40%; AUC = 0.86; positive predictive value [PPV] = 100%). Notably, combining *CYFRA 21‐1*, *CEA*, *ProGRP* and neuron‐specific enolase (*NSE*) significantly improved SCLC diagnostic accuracy beyond individual biomarkers.

Similarly, Korkmaz and co‐workers measured serum levels of six tumour markers (*ProGRP*, squamous cell carcinoma antigen [*SCCAg*], *CYFRA 21‐1*, *HE4*, chromogranin A [CgA] and *NSE*) in 99 lung cancer patients and 30 benign pulmonary disease controls using mass spectrometry [47]. *ProGRP* concentrations were significantly elevated in SCLC (*p* = 0.009), while *CYFRA 21‐1* and *SCCAg* showed higher expression in NSCLC (*p* = 0.019 and *p* = 0.001, respectively). *CYFRA 21‐1* (*p* < 0.001; *r* = 0.394), *HE4* (*p* = 0.014) and *CgA* (*p* = 0.023) levels positively correlated with NSCLC stage progression. Among all markers, *ProGRP* demonstrated optimal performance in differentiating histological subtypes (AUC = 0.875).

In parallel, Wen and colleagues evaluated a 10‐biomarker panel to establish diagnostic profiles for lung cancer subtypes in 250 serum samples [48]. *CEA* showed optimal efficacy for adenocarcinoma (AUC = 0.812; sensitivity = 63.9%), while *CYFRA 21‐1* (AUC = 0.847; sensitivity = 84.6%) and *CEA* (AUC = 0.804; sensitivity = 70.0%) were most effective for squamous cell carcinoma. For SCLC, *NSE* (AUC = 0.819; sensitivity = 69.0%) and *CEA* (AUC = 0.808; sensitivity = 60.7%) achieved robust diagnostic performance.

Supporting these findings, Sua and co‐authors analysed five serum biomarkers in 93 pulmonary disease patients [49]. Receiver operating characteristic (ROC) curves revealed significantly elevated median levels of *CYFRA 21‐1*, *SCC‐Ag*, *ProGRP*, *CEA* and *NSE* in malignant versus benign groups, underscoring their utility for histological classification prior to tissue confirmation.

#### 2.3.2 Exosomes and Other Proteomics‐Based Biomarkers

Recent advances position exosomes as promising tumour biomarkers. Sun and colleagues conducted label‐free quantitative proteomic analyses of serum and salivary exosomes from healthy individuals and patients with lung cancer, identifying 11 significantly dysregulated proteins with diagnostic potential and confirming the presence of disease‐associated proteins in exosomes [50]. Proteomic studies further suggest autoantibodies against α‐enolase as potential NSCLC biomarkers; when combined with carcinoembryonic antigen (*CEA*) and *CYFRA 21‐1*, they substantially enhanced diagnostic sensitivity [51]. Similarly, Patz and co‐workers profiled serum proteins from 100 lung cancer patients, identifying a diagnostic panel (*CEA*, retinol‐binding protein [*RBP*], squamous cell carcinoma antigen [*SCC*], alpha‐1‐antitrypsin [*A1AT*]) with high accuracy [52].

Salivary proteomics also shows promise: Jiang and colleagues analysed samples from 89 early‐stage lung cancer patients, 11 advanced cases and 50 healthy controls, developing a diagnostic platform with high sensitivity and specificity for early detection [53]. Parallel efforts by Pan and co‐authors established a serum‐based protein panel (*p53*, *HRas*, *ETHE1*) to facilitate early diagnosis, with 50% sensitivity at > 90% specificity [54]. While early NSCLC detection remains challenging, proteomics offers compelling biomarker discovery avenues. In serum studies, paraoxonase 1 (*PON1*) emerged as a potential stage I biomarker following glycopeptide enrichment with N‐acetylglucosamine (*GlcNAc*)‐binding lectins; combined AANL‐enriched PON1 and AANL‐enriched AACT were significantly different between early NSCLC samples and tumour‐free samples with an AUC of 0.940, 94.4% sensitivity and 90.2% specificity [55]. Comparative tissue proteomics identified dysregulated cathepsin D (*CTSD*) and heat shock protein 60 (*HSP60*) as candidates for early lung squamous cell carcinoma (*LSCC*) detection [56].

Notably, Ahn and colleagues used multiple reaction monitoring (MRM) mass spectrometry to identify fucosylated proteins (*APCS*, *C9*, *SERPINA4*, *PON1*) in SCLC [57]. Despite decreased PON1 levels, its fucosylation increased—highlighting a diagnostically relevant post‐translational modification. Separately, Guergova‐Kuras and co‐workers applied monoclonal antibody‐based proteomics to detect five high‐potential protein candidates across NSCLC cohorts [58]. Combining CYFRA, an established cancer marker, with the panel resulted in a performance of 83% sensitivity at 95% specificity for stage I NSCLC.

### 2.4 Metabolomics and Volatilomics

#### 2.4.1 Metabolomics

Lactate accumulation represents a common metabolic alteration in lung cancer, where tumour cells preferentially metabolise glucose to lactate even under oxygen‐sufficient conditions—a phenomenon termed the Warburg effect [59]. Phospholipids, as key components of cell membranes, frequently exhibit dysregulated metabolism in lung cancer [60]. Reflecting this, altered phospholipid profiles are consistently reported in patient plasma. Yu and colleagues used liquid chromatography‐mass spectrometry (LC–MS) to identify elevated lysophosphatidylethanolamine (LPE 18:1) and phosphatidylethanolamine (PE 40:4), alongside reduced cholesteryl ester (ce 18:2) and sphingomyelin (SM 22:0), in lung cancer patients versus healthy controls. A classifier based on these phospholipids achieved area under the curve (AUC) values of 0.823 and 0.808 in training and validation cohorts, respectively [61]. Similarly, an LC–MS analysis of 100 early‐stage lung cancer patients and 300 controls revealed increased phosphatidylcholine (PC), diacylglycerols and sphingomyelin, but decreased lysophosphatidylcholine species (LPC 18:2, 18:1, 18:0). A seven‐phospholipid panel derived from these alterations yielded an AUC of 0.88 [62].

Notably, a non‐targeted lipidomics study of 311 participants identified nine phospholipids as diagnostic features for early lung cancer. A targeted LC–MS model developed from this signature achieved 100% specificity in independent validation, with ≥ 90% sensitivity and 92% specificity in a larger cohort of 1036 individuals undergoing LDCT screening and 109 prospective clinical samples [63].

Beyond phospholipids, dysregulated urinary creatine and creatinine levels show promise as early diagnostic biomarkers [64]. A prospective LC–MS analysis of 178 lung cancer patients and 351 healthy controls demonstrated robust correlations between elevated urinary creatine concentrations and lung cancer risk across European and non‐European populations [65]. Significantly, upregulated creatine and creatinine levels were also detected in serum and saliva from affected individuals, supporting their utility as biomarkers across multiple biofluids [53].

#### 2.4.2 Volatilomics

Volatile organic compounds (VOCs)—gaseous organic chemicals detectable at room temperature—show promise as targets for early lung cancer detection, with carbonyl‐containing VOCs in exhaled breath actively investigated as diagnostic biomarkers [66]. Using Fourier‐transform ion cyclotron resonance mass spectrometry (FTICR‐MS), Fu and colleagues identified significantly elevated breath levels of 2‐butanone, 2‐hydroxyacetaldehyde, 3‐hydroxy‐2‐butanone and 4‐hydroxyhexenal (4‐HHE) in lung cancer patients versus healthy controls and individuals with benign pulmonary nodules [66]. Bousamra and co‐workers further demonstrated that diagnostic accuracy directly correlates with the number of elevated VOC markers: detection of ≥ 3 elevated markers achieved 0.95 specificity in distinguishing lung cancer from healthy controls [67]. Building on this, a diagnostic model incorporating six carbonyl VOCs (these four compounds plus acrolein and malondialdehyde) effectively discriminated lung cancer patients from healthy individuals and showed moderate discrimination against benign nodules. This model achieved ≥ 0.96 sensitivity with specificity ranging from 0.64 (benign nodule cohort) to 1.00 (non‐smokers) across study populations [68].

### 2.5 Microbiome Signatures

#### 2.5.1 Airway Microbial Biomarkers

Emerging research highlights the diagnostic potential of the lung cancer microbiome. Lee and colleagues compared 20 patients with lung cancer to eight with benign lesions, identifying elevated *Veillonella* and *Megasphaera* in the cancer cohort. Their combined microbial signature achieved an AUC of 0.88, with 0.95 sensitivity and 0.75 specificity [69]. Similarly, Bello and co‐workers demonstrated that *Streptococcus* alone yielded 0.897 diagnostic accuracy [70]. Jin and co‐authors conducted metagenomic sequencing of 91 lung cancer patients, 29 non‐malignant disease patients and 30 healthy individuals, identifying 11 differential bacterial genera (AUC = 0.796). Subsequently, Cheng and colleagues integrated tumour markers (*CEA*, *NSE*, *CYFRA21‐1*) with bacterial biomarkers (*Pseudomonadaceae*, *Gemmiger*, candidate phylum TM7‐3) to establish a combined diagnostic model achieving an AUC of 0.84 [71].

#### 2.5.2 Gut Microbial Biomarkers

Zhang and colleagues reported significant faecal enrichment of *Bacteroidetes*, *Veillonella* and *Clostridium* in lung cancer patients versus healthy controls (41 per group) [72]. Building on this, Zheng and co‐workers validated in discovery and validation cohorts that gut microbiota β‐diversity markedly differed between lung cancer patients and healthy individuals [73]. A diagnostic model based on 13 operational taxonomic units (OTUs) achieved an AUC of 0.976 in the discovery cohort, though performance declined to 0.764 in independent validation (34 lung cancer vs. 40 controls). While these findings indicate diagnostic potential, larger multi‐centre studies and mechanistic investigations are needed to establish clinical utility.

#### 2.5.3 Blood Microbial Biomarkers

A study of 58 NSCLC patients and 58 healthy controls used digital droplet PCR (ddPCR) to detect elevated *Selenomonas*, *Streptococcus* and *Veillonella* in blood samples. The diagnostic model achieved 0.75 sensitivity and 0.78 specificity, with consistent performance in an independent validation cohort (93 per group) [74]. Chen and colleagues conducted whole‐genome sequencing of plasma from 69 lung cancer patients and 97 healthy individuals, revealing a distinct microbial species composition despite a marginally lower proportion of microbial reads in cancer samples (0.009% vs. 0.012%) [75]. Their model, based on enriched microbial taxa, achieved an AUC of 0.95 (0.81 sensitivity, 0.90 specificity) in training and maintained robust performance in two independent validation cohorts (AUCs 0.93–0.921), supporting blood microbiome signatures as a non‐invasive approach for early detection.

Collectively, these findings are synthesised in Table 1, which summarises representative multi‐omics biomarkers for early‐stage lung cancer.

**TABLE 1:** Multi‐omics biomarkers for early‐stage lung cancer detection.

Omics type	Sample source	Representative biomarkers	Study design	External validation	Diagnostic performance (AUC/sensitivity/specificity)	References
Genomics	BAL, plasma, tumour tissue	EGFR, KRAS, BRAF, HER2, ALK, RET, MET	Case–control	No	Combined mutation panels show higher sensitivity than single genes; e.g., p53 mutation sensitivity 56% in BAL	[10, 11]
Epigenomics (DNA methylation)	Sputum, plasma, BAL	SHOX2, RASSF1A, PTGER4, TAC1, HOXA17, SOX17, PCDHGB6, miR‐126	Prospective (sputum studies), Case–control	Partial (some panels externally validated)	Sensitivity up to 98%, Specificity ~81% for multi‐gene panels	[21, 22, 23, 24, 25, 76, 77]
Transcriptomics (miRNA)	Serum, plasma, exosomes	miR‐20a, miR‐21, miR‐145, miR‐223, miR‐375, miR‐3149, miR‐4769‐3p	Case–control	Yes (Nadal 4‐miRNA panel externally validated)	AUC up to 0.993 for miRNA panels, e.g., Nadal panel (4‐miRNA); individual AUCs from 0.73 to 0.94	[31, 32, 33, 34, 35, 36, 37, 38, 78, 79, 80, 81]
Non‐coding RNAs (lncRNA, circRNA)	Tumour tissue, plasma, saliva, exosomes	lncRNA HOTAIR, GAS5; circRNA hsa_circ_0077837, hsa_circ_0001821	Case–control	Partial (circRNA panels validated in some independent cohorts)	AUC for circRNA meta‐analysis up to 0.78 in Chinese cohort	[42, 43, 44, 45, 82, 83]
Proteomics	Serum, saliva, exhaled breath condensate	CEA, CYFRA 21‐1, ProGRP, NSE, SCCAg, HE4, CTSD, SCCA1/2, PON1, HSP60	Case–control, Prospective serum studies	Yes (some panels validated externally)	AUC up to 0.94 (GEP panel with CEA, NSE, CYFRA21‐1); ProGRP for SCLC (AUC = 0.875)	[45, 47, 48, 49, 84, 85]
Exosomal proteins	Serum, saliva	CD9+, CD81+ enriched markers (e.g., A1AT, RBP, ERO1L, PABPC4)	Case–control	Partial	Some panels show > 80% sensitivity/specificity	[50, 52, 86]
Metabolomics (serum)	Plasma	LPE 18:1, PE 40:4, CE 18:2, SM 22:0, PC, LPCs	Prospective	Yes	7‐metabolite model AUC = 0.88; Sensitivity ~90%, Specificity ~92%	[61, 62, 63]
Metabolomics (urine)	Urine	Creatinine, creatine, uric acid	Prospective	Yes	Creatinine level elevation correlates with risk across ethnic groups	[53, 65, 87]
Volatilomics	Breath	2‐butanone, 3‐hydroxy‐2‐butanone, 4‐HHE, acrolein	Case–control	Partial	VOC panel sensitivity ≥ 96%, specificity up to 100% in non‐smokers	[66, 67, 68]
Airway microbiome	BAL, bronchial brushing	Veillonella, Megasphaera, Streptococcus	Case–control	Partial	AUC = 88%–98%, Sensitivity > 90% with ML‐based models	[69, 70, 71]
Gut microbiome	Stool	Bacteroidetes, Clostridium, Veillonella	Case–control	Yes (external validation cohort)	Discovery model AUC = 97.6%; Validation AUC = 76.4%	[72, 73]
Blood microbiome	Plasma	Selenomonas, Streptococcus, Veillonella	Case–control	Yes	ML models AUC = 93%–95% in validation cohorts	[74, 75]

## 3 AI‐Driven Integration of Multi‐Omics Data

### 3.1 Machine Learning for Biomarker Discovery

#### 3.1.1 Cancer Biomarker Discovery Using Machine Learning

Lung adenocarcinoma (LUAD) and lung squamous cell carcinoma (LUSC) constitute the two most prevalent histological subtypes. While conventional treatments overlap, profound molecular differences justify their classification as distinct entities [88]. Computationally, both early detection and histological subtyping represent classification tasks—a machine learning framework successfully applied across pan‐cancer datasets with significant diagnostic value. From a methodological perspective, machine learning approaches for lung cancer biomarker discovery can be broadly categorised into supervised, unsupervised and multimodal learning frameworks, each addressing distinct analytical objectives. Supervised learning models, which rely on labelled outcomes such as LUAD versus LUSC or malignant versus benign status, are most commonly used for early detection and histological subtyping. In imaging‐based biomarker discovery, convolutional neural networks (CNNs) have been extensively applied to radiomic feature learning from CT scans, enabling automated extraction of tumour shape, texture and intensity patterns and linking these features to molecular programmes and clinical outcomes [89]. In contrast, unsupervised learning approaches aim to uncover latent molecular structures without predefined labels and are particularly suited for high‐dimensional omics data. Autoencoder‐based architectures, including variational autoencoders, have been widely used to compress genomic or epigenomic profiles into biologically meaningful latent representations and to identify molecularly distinct cancer subtypes [90]. In addition to conventional machine learning models, deep learning architectures explicitly tailored for multi‐omics integration have shown promise for cancer subtype classification and biomarker discovery, leveraging attention mechanisms or graph structures to model cross‐modal dependencies. For example, self‐attention based deep learning networks integrate heterogeneous omics layers by learning joint latent representations that capture inter‐omics relationships across samples, outperforming concatenation‐based approaches in cancer subtype recognition tasks [91]. Similarly, graph‐based neural networks that combine multi‐omics data with graph autoencoder or graph attention mechanisms have been proposed to capture complex interactions among omics features and improve subtype clustering and classification [92]. These multimodal frameworks demonstrate how structured deep learning models can integrate genomic, epigenomic and transcriptomic features into unified representations for improved diagnostic performance. Jiao et al. developed a deep learning model to classify 24 common cancer types using somatic passenger mutation profiles from 2606 whole‐genome sequenced tumours. The model reached 91% accuracy on unseen tumours and 88%–83% on independent primary and metastatic samples [93, 94].

Somatic mutations (single‐nucleotide variants, insertions, deletions) exhibit cancer‐type specificity and serve as valuable classification features [95]. Driver mutations further shape distinctive gene expression patterns [96, 97] enabling RNA sequencing‐based models to discriminate malignant from benign tumours and differentiate LUAD from LUSC [98]. Copy number variations (CNVs), another common genomic aberration, enhance classification accuracy when integrated into models [99]. For example, Daemen and colleagues developed a recursive hidden Markov model to precisely identify CNV regions. Across two public array‐CGH cohorts and an internal ovarian cancer set, patient subgroups were defined and recurrent HMMs were applied to detect subtype‐specific chromosomal alterations. After reducing these regions through multiple univariate selection approaches, a weighted LS‐SVM classifier was trained to handle data imbalance, achieving 88%–95.5% accuracy in cross‐validation [98]. In a prospective multi‐centre study, bronchial epithelial cells from 299 smokers were profiled using RNA microarrays to train a logistic‐regression–based gene‐expression model for lung cancer detection. From 232 cancer‐associated transcripts, a 17‐gene classifier incorporating smoking variables, sex and age was developed. It achieved an AUC of 0.78 in patients with non‐diagnostic bronchoscopy and showed comparable performance in an independent cohort (AUC 0.81), with a negative predictive value of 94% [100]. Using multiple cohorts and next‐generation sequencing, Yoonha Choi et al. built a Genomic Sequencing Classifier (GSC) by integrating clinical‐ and genomic‐focused ensemble models, hierarchical regression to remove major clinical effects before fitting genomic signals and selective clinical–genomic interaction terms to reduce confounding. The final model incorporated 1232 genes and four clinical variables. In 412 validation samples, it reclassified low‐ and intermediate‐risk individuals with 45% specificity and 91% sensitivity (NPV 95%), and further identified subsets with elevated post‐test risk, achieving PPVs of 65% and 91% in the intermediate‐ and high‐risk groups, respectively [96]. To mitigate overfitting, studies frequently employ feature selection methods including LASSO, recursive feature elimination and univariate filtering [97, 100, 101].

Integration of multi‐omics data with clinical information substantially improves model performance, particularly within multimodal architectures that combine modality‐specific encoders with downstream fusion layers, enabling complementary biological signals to be captured while reducing noise and redundancy [96, 98, 102]. Diverse algorithms—from support vector machines and random forests to gradient boosting and neural networks—have proven effective for lung cancer detection and classification. Deep learning on genomic data faces issues such as extreme feature dimensionality, limited sample size, poor interpretability and lack of inherent structure. To address these, Kazuma Kobayashi et al. adapted Diet Networks by incorporating element‐wise input scaling to further reduce parameters and stabilise training. Tested on 950 lung cancer cases (adenocarcinoma vs. squamous carcinoma) using 5‐fold cross‐validation, the model reached ~80% accuracy and yielded latent features that clarified mutation–prediction relationships [95].

Deep learning shows particular promise for biomarker discovery. Xiao and co‐workers identified informative genes through differential expression analysis, developing an ensemble model incorporating five classifiers that significantly outperformed conventional methods in tumour detection. Their deep learning‐based multi‐model ensemble method achieved a prediction accuracy of 99.20% on the LUAD dataset, with an AUC of 0.988 [103]. Mohammed and colleagues created a deep learning framework for multi‐cancer classification using RNA‐seq data, combining synthetic minority oversampling with undersampling to address class imbalance; their approach demonstrated robust generalisation across cancer types. The results showed that, with or without LASSO, the ensemble approach outperformed the other classifiers. In addition, multiple low‐sampling traditional methods (including various SVMs, ANN, KNN and bagged trees) performed better than their high‐sampling counterparts [104]. For early prediction, Liu and co‐authors designed a deep neural network leveraging Kullback–Leibler divergence and focal loss, achieving an AUC of 0.99—outperforming traditional methods [105]. Wang and colleagues further developed an unsupervised variational autoencoder using epigenetic methylation data to differentiate LUAD from LUSC, achieving near‐perfect discrimination (AUC ≈1.0) and highlighting the diagnostic value of epigenetic features [106].

### 3.2 Clinical Decision Support Systems

Precision oncology advances have enabled extensive application of multi‐omics technologies—including genomics, transcriptomics, pathology and radiomics—to cancer diagnosis and subtyping. Artificial intelligence (AI) algorithms integrate these large‐scale datasets, enhancing diagnostic accuracy and cancer classification [107, 108].

Deep neural network (DNN) models achieve high accuracy in malignancy identification, particularly when analysing digitised histopathological slides to distinguish tumour tissue. For example, an AI system (HMS and MITII) detecting breast cancer metastases in haematoxylin and eosin (H&E)‐stained lymph nodes (*n* = 270) achieved an AUC of 0.994, outperforming pathologists (AUC 0.810) [109]. Using TCGA whole‐slide images from lung adenocarcinoma and squamous cell carcinoma cases (*n* = 884), Yu et al. trained convolutional neural networks to analyse histopathology and validated performance in an external cohort. The models accurately distinguished tumour from benign tissue and reproduced pathologist‐level classification, while quantitative image features were strongly associated with major transcriptomic subtypes (*p* < 0.01). This fully automated framework enables objective identification of molecular subtypes in non‐small cell lung cancer without prior pathological annotation [5, 110]. Coudray and colleagues developed an algorithm classifying The Cancer Genome Atlas (TCGA) lung cancer slides into adenocarcinoma, squamous carcinoma and normal tissue (AUC 0.97), while simultaneously predicting mutations in key driver genes (*STK11*, *EGFR*, *KRAS*, *TP53*) through pathological feature integration [111]. Subsequent studies confirm AI's capability to predict genetic alterations and microsatellite instability [112]. Using transfer learning on over 17,355 H&E slides from 28 tumour types, Fu et al. extracted computational histopathology features that accurately distinguish cancers and tumour regions, and show strong associations with genomic alterations, gene expression patterns, immune infiltration and patient prognosis. These results demonstrate the capacity of computer vision to link tissue morphology with the molecular and clinical landscape of cancer [113].

Radiological techniques—including magnetic resonance imaging (MRI), computed tomography (CT) and positron emission tomography‐CT (PET‐CT)—are cornerstone diagnostic modalities in oncology. CNN‐based algorithms have demonstrated remarkable diagnostic accuracy for tumours using these imaging sources [114]. Models trained on MRI data, for instance, can distinguish malignant tumours from benign lesions. For instance, using 2D T2‐weighted prostate MRI from 172 patients, a deep convolutional neural network was compared with a SIFT–BoW–based approach for automated prostate cancer detection. The deep learning model achieved significantly higher diagnostic performance (AUC 0.84) than the non‐deep method (AUC 0.70, *p* < 0.001), demonstrating its superiority for distinguishing prostate cancer from benign conditions and its potential applicability to other imaging modalities [115]. Similarly, in a retrospective analysis of nearly 30,000 mammograms, McKinney and colleagues developed an AI system that outperformed multiple radiologists in diagnostic AUC for breast cancer [116].

Beyond medical imaging, multi‐omics data are increasingly used for cancer diagnosis, classification and grading. The evolution of high‐throughput omics technologies is shifting cancer classification from histopathological approaches toward molecular taxonomy [117]. For example, Sun and colleagues developed a genomics deep learning (GDL) model using DNNs trained on whole‐exome sequencing (WES) data from The Cancer Genome Atlas (TCGA), encompassing 12 cancer types and 1991 healthy samples. This model achieved high diagnostic accuracy (AUC = 0.94) in distinguishing cancer from normal tissue [118]. Similarly, Capper and colleagues employed machine learning to analyse DNA methylation profiles for tumour classification, demonstrating diagnostic performance comparable to pathologist assessments—and surpassing them in diagnostically challenging cases. They show that the availability of this method may have a substantial impact on diagnostic precision compared to standard methods, resulting in a change of diagnosis in up to 12% of prospective cases [119]. Subsequent studies have validated machine learning classifiers based on DNA methylation and copy number variation in diagnostic settings (*n* > 2000) [120].

While AI‐driven multi‐omics models have demonstrated strong diagnostic performance across imaging and molecular domains, their clinical impact ultimately depends on integration into established lung cancer screening and surveillance workflows. LDCT remains the foundation of population‐based lung cancer screening but is limited by high false‐positive rates and a large proportion of indeterminate pulmonary nodules. In this context, multi‐omics AI systems are best positioned as clinical decision support tools that complement LDCT by refining malignancy risk rather than replacing radiologic assessment. Several clinically validated platforms illustrate this translational pathway. For example, airway‐based gene expression classifiers such as the Percepta Genomic Sequencing Classifier use machine learning to re‐stratify cancer risk following nondiagnostic bronchoscopy, enabling both down‐classification of low‐risk nodules and escalation of high‐risk cases, thereby reducing unnecessary invasive procedures [121, 122]. Blood‐based multi‐omics approaches provide additional orthogonal information; the Galleri multi‐cancer early detection test integrates cell‐free DNA methylation patterns using machine learning and has demonstrated high specificity in large validation cohorts, supporting its potential role as an adjunct to LDCT in risk refinement [123]. Beyond single time‐point assessment, longitudinal integration of circulating tumour DNA or methylation signals into AI‐driven surveillance algorithms offers a framework for dynamic risk updating in high‐risk individuals or patients with stable nodules. Importantly, several of these technologies are being evaluated in prospective, multicentre studies (e.g., CCGA and PATHFINDER) designed to assess real‐world feasibility and clinical utility. Collectively, these examples highlight a realistic model in which multi‐omics AI systems function as embedded decision support tools within LDCT‐based screening and surveillance pathways, enhancing risk stratification, optimising downstream management and facilitating earlier and more precise lung cancer detection.

To clarify AI's role in integrating multi‐omics data for early lung cancer diagnosis, Figure 1 outlines a schematic workflow from raw data processing to clinical interpretation. Complementing this, Table 2 summarises exemplary diagnostic implementations of AI‐driven multi‐omics integration. Together, these approaches show significant potential for enhancing precision in cancer diagnosis, classification and grading.

**TABLE 2:** AI applications in multi‐omics data integration for early lung cancer detection.

Application focus	AI methods used	Integrated data types	Reported benefits	References & section
Biomarker discovery	Random Forest, SVM, LASSO	Genomics+Epigenomics (e.g., methylation+mutation)	Improved feature selection and early detection sensitivity via combinatorial markers	[7, 95, 101, 124]
Molecular subtyping	Deep Neural Network (DNN), k‐NN	Transcriptome+Methylome	Accurate LUAD/LUSC classification; robust clustering of expression and epigenetic profiles	[95]
Early diagnosis modelling	CNN, Autoencoder, Logistic Regression	ctDNA+miRNA+Proteomics+Clinical features	AUC > 0.98 in some multi‐omics models (e.g., CNN integrating 4‐omics in lung nodules classification)	[104, 106]
Risk stratification	Hierarchical Clustering+ML ensemble	Methylation+Gene expression+miRNA	Separation of high−/low‐risk subgroups for early‐stage NSCLC patients	[112, 125, 126]
Treatment response prediction	Gradient Boosting, GNN, Transfer Learning	RNA‐seq+Immune signatures+Histology	AI models can predict immune checkpoint inhibitor response with high accuracy (AUC > 0.9)	[127, 128]
Survival prediction	Cox Proportional Hazards+DNN	Clinical+Transcriptomic+Histopathologic image	Integration improves C‐index; model interpretable via SHAP and attention‐based layers	[129, 130]
Radiogenomics	Multi‐task learning, XGBoost, FusionNet	Imaging+Genomics+Clinical	Combines PET‐CT with genomics to infer mutations (e.g., EGFR status) without biopsy	[131]
Multi‐modal fusion models (computational architectures that integrate multiple data modalities)	Autoencoder+DNN+Ensemble Fusion	Genomics+Proteomics+Metabolomics+Clinical	Unified latent space improves classification performance and reduces noise from individual omics layers	[118]
Decision support systems	Explainable AI, Knowledge Graphs	Omics+EHR+Imaging	Clinical workflow integration, enables transparent diagnosis and treatment recommendation	[132, 133, 134]

## 4 Challenges and Future Directions

### 4.1 Technical Challenges

#### 4.1.1 Data Heterogeneity and Integration Challenges

Multi‐omics data—spanning genomics, transcriptomics, proteomics, metabolomics and epigenomics—originate from diverse analytical platforms, resulting in substantial heterogeneity in data formats, resolutions, scales and quality. Effective integration requires robust standardisation protocols, batch effect correction and advanced harmonisation methods. Inadequate preprocessing risks overfitting or loss of critical biological signals [135, 136].

#### 4.1.2 Scarcity of High‐Quality Annotated Datasets

Current studies often rely on single‐institution cohorts with limited racial and demographic diversity. Public datasets frequently exhibit incomplete clinical annotations, inconsistent phenotyping, or bias toward advanced‐stage cancers. The scarcity of large‐scale, multicentre early‐stage lung cancer repositories hinders AI generalisability and robust biomarker discovery [137].

#### 4.1.3 Limited AI Model Interpretability

State‐of‐the‐art deep learning models often operate as “black boxes,” obscuring the biological mechanisms underlying their predictions. This interpretability deficit hampers clinical translation, regulatory approval and mechanistic validation of biomarkers. Although explainable artificial intelligence (XAI) methods are emerging, their adoption in lung cancer research remains nascent [138]. Several specific XAI techniques have been explored to enhance transparency and clinical trust. Gradient‐weighted Class Activation Mapping (Grad‐CAM), for example, generates spatial heatmaps that highlight regions of input imaging data—such as CT scans—that most strongly influence model decisions, allowing clinicians to visually inspect whether the model focuses on medically relevant structures rather than confounding artefacts; this approach has been applied in lung cancer classification models integrating deep convolutional networks with Grad‐CAM visualisations to improve interpretability (e.g., CT imaging studies incorporating Grad‐CAM to visualise salient features for subtype differentiation) [139]. Local Interpretable Model‐agnostic Explanations (LIME) and SHapley Additive exPlanations (SHAP) values have also been used in recent lung cancer AI frameworks to quantify feature contributions: LIME perturbs input features to build locally faithful interpretable approximations of complex models, while SHAP, grounded in game theory, provides global and local measures of feature importance that can identify which clinical, radiomic, or molecular input variables drive predictive outputs [140]. For instance, hybrid deep learning models for lung cancer detection have integrated SHAP to interpret the influence of specific features on classification outcomes, thereby linking model predictions to biologically plausible determinants and supporting clinician understanding of risk factors [141]. By combining these XAI approaches, researchers can move beyond simple performance metrics to generate interpretable evidence that increases clinician confidence, facilitates mechanistic insight and supports regulatory evaluation, ultimately fostering more trustworthy AI‐assisted tools in early lung cancer diagnosis.

#### 4.1.4 Reproducibility and Standardisation Deficits

The lack of standardised protocols for data preprocessing, feature selection and model evaluation undermines reproducibility. Establishing standardised reporting frameworks, developing robust benchmark datasets and creating open‐source analytical pipelines are essential for field‐wide advancement [142, 143].

#### 4.1.5 Clinical Workflow Integration Barriers

Most multi‐omics AI tools remain confined to preclinical development and are not integrated into clinical decision support systems. Routine implementation requires solutions to operational challenges, including cost constraints, analytical turnaround time, interpretable outputs and interoperability with electronic health records (EHRs).

#### 4.1.6 Regulatory and Ethical Complexities

Deploying AI with sensitive biomedical data raises critical ethical concerns, including patient privacy, data governance and dynamic consent frameworks. Evolving regulations for AI diagnostics and multi‐omics integration create approval uncertainties that may delay clinical adoption.

### 4.2 Clinical Translation

Translating multi‐omics discoveries and AI‐derived biomarkers into practice remains the primary challenge in early lung cancer detection. While advances in molecular signatures and predictive algorithms are significant, their implementation faces persistent scientific, regulatory, infrastructural and clinical barriers. Future innovation must prioritise feasible, reliable and patient‐centred translational pathways.

To ensure clinical utility, research should shift from exploratory omics toward standardised biomarker panels with rigorous diagnostic validation. These panels require high sensitivity and specificity across diverse populations and contexts—particularly for high‐risk asymptomatic individuals. Large‐scale prospective trials are essential to evaluate real‐world performance in screening programmes.

AI models must progress beyond experimental prototypes to interpretable, regulatory‐compliant tools with seamless electronic health record (EHR) integration and clinical decision support. Development should prioritise explainable artificial intelligence (XAI) to enhance transparency, foster clinician trust and improve adoption. Critical capabilities include real‐time processing, continuous model refinement and intuitive human–AI interfaces for workflow integration.

Successful translation necessitates diverse, high‐quality datasets from international multicentre collaborations. Systematic curation of multi‐omics profiles, imaging data, clinical parameters and longitudinal outcomes across multiethnic cohorts is fundamental. Harmonised protocols for data acquisition and metadata annotation will ensure reproducibility and generalisability.

Concurrently, evolving regulatory frameworks demand proactive collaboration with authorities to establish evidence‐based standards for data quality, model interpretability and clinical safety. Ethical imperatives—including dynamic consent, data privacy preservation and bias mitigation—must be addressed to ensure equitable implementation. The ultimate objective is personalised screening frameworks integrating multi‐omics biomarkers, AI‐driven risk stratification and longitudinal monitoring. This evolution from population‐based to precision prevention enables earlier detection when intervention is most effective.

### 4.3 Emerging Opportunities in AI‐Driven Multi‐Omics Integration

The rapid convergence of high‐throughput multi‐omics technologies and advanced artificial intelligence (AI) is creating unprecedented opportunities for early lung cancer detection. These advances offer both enhanced diagnostic precision and a transformative shift toward predictive, preventive precision oncology, presenting key opportunities to reshape diagnostic paradigms.

#### 4.3.1 Single‐Cell Multi‐Omics for Early Lesion Characterisation

Conventional bulk omics approaches obscure cellular heterogeneity in early lung lesions. Single‐cell multi‐omics technologies—particularly integrated single‐cell RNA sequencing (scRNA‐seq) and single‐cell assay for transposase‐accessible chromatin sequencing (scATAC‐seq)—enable comprehensive profiling of pre‐cancerous states, tumour microenvironment dynamics and clonal evolution at cellular resolution. These methods identify rare pre‐malignant subpopulations and reveal initial molecular alterations preceding tumour formation. With decreasing costs and improved scalability, single‐cell analysis is increasingly integral to early biomarker discovery.

#### 4.3.2 Liquid Biopsy Multi‐Omics Integration

Circulating biomarkers—including circulating tumour DNA (ctDNA), extracellular vesicles, exosomes and cell‐free RNA (cfRNA)—represent critical non‐invasive diagnostic resources. Integrative analysis of DNA methylation, proteomic and metabolomic signatures from liquid biopsies enhances sensitivity and specificity for early detection. Coupling longitudinal liquid biopsy data with AI‐driven risk models enables dynamic disease surveillance and timely intervention.

#### 4.3.3 Privacy‐Preserving AI via Federated Learning

Multisite biomarker research encounters substantial data‐sharing barriers. Federated learning overcomes this challenge by facilitating collaborative artificial intelligence model training across institutions without exchanging raw patient data. Applied to multisite lung cancer cohorts, this approach accelerates biomarker validation while ensuring compliance with patient privacy requirements.

#### 4.3.4 Radiogenomic‐Spatial Omics Fusion

Radiogenomics represents an integrative approach that links quantitative imaging features extracted from radiological modalities (e.g., CT or PET) with underlying genomic or transcriptomic profiles, aiming to infer tumour molecular characteristics from imaging phenotypes. Advances in spatial transcriptomics and proteomics now enable molecular cartography of early lesions in situ. Computational integration of spatial multi‐omics with low‐dose CT enhances diagnostic subtyping and precise lesion Localisation.

#### 4.3.5 Dynamic AI Monitoring and Digital Twins

**FIGURE 2:**
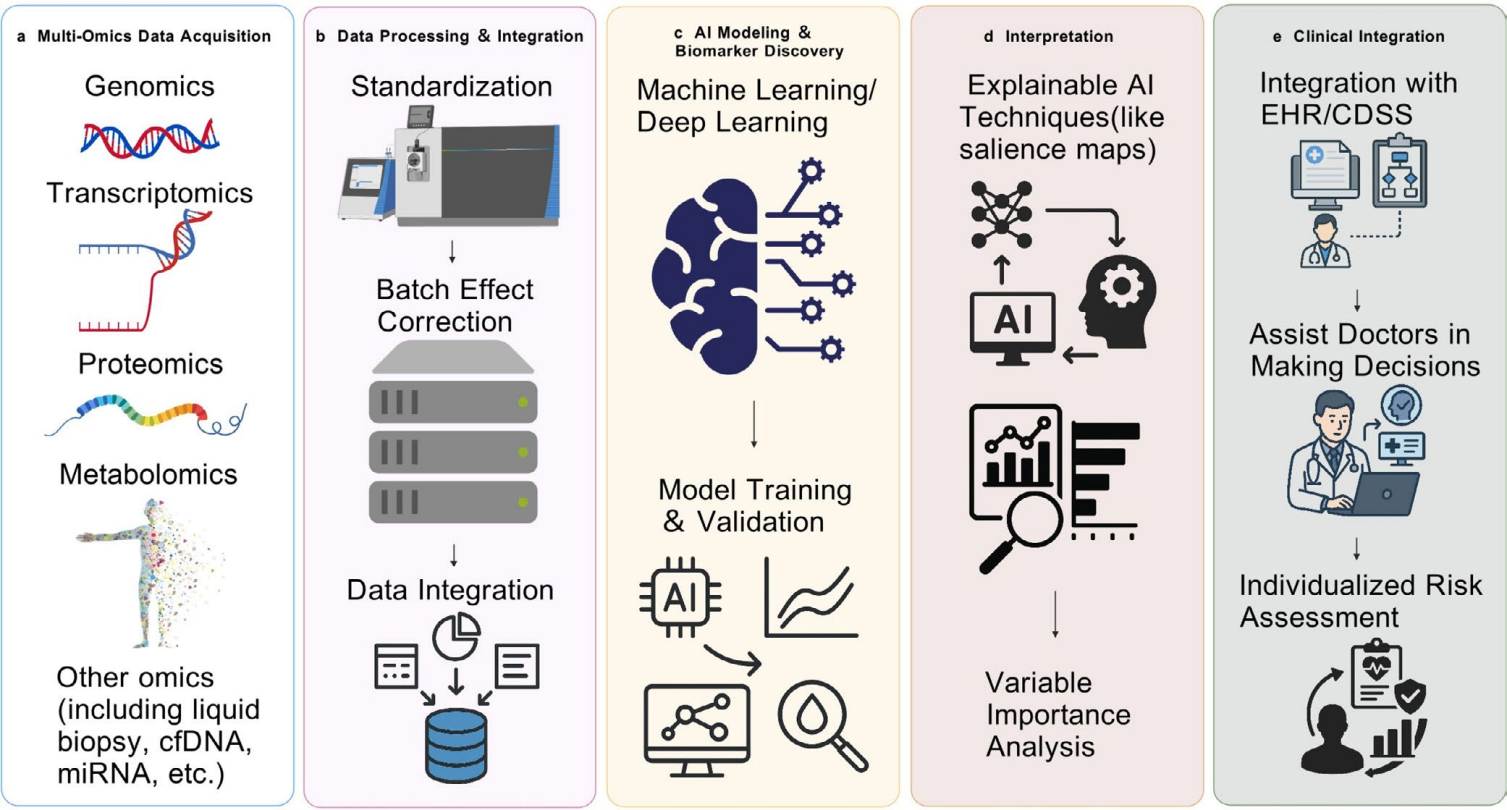
AI‐driven multi‐omics workflow for early lung cancer detection. Generated using BioGDP.com.

**FIGURE 3:**
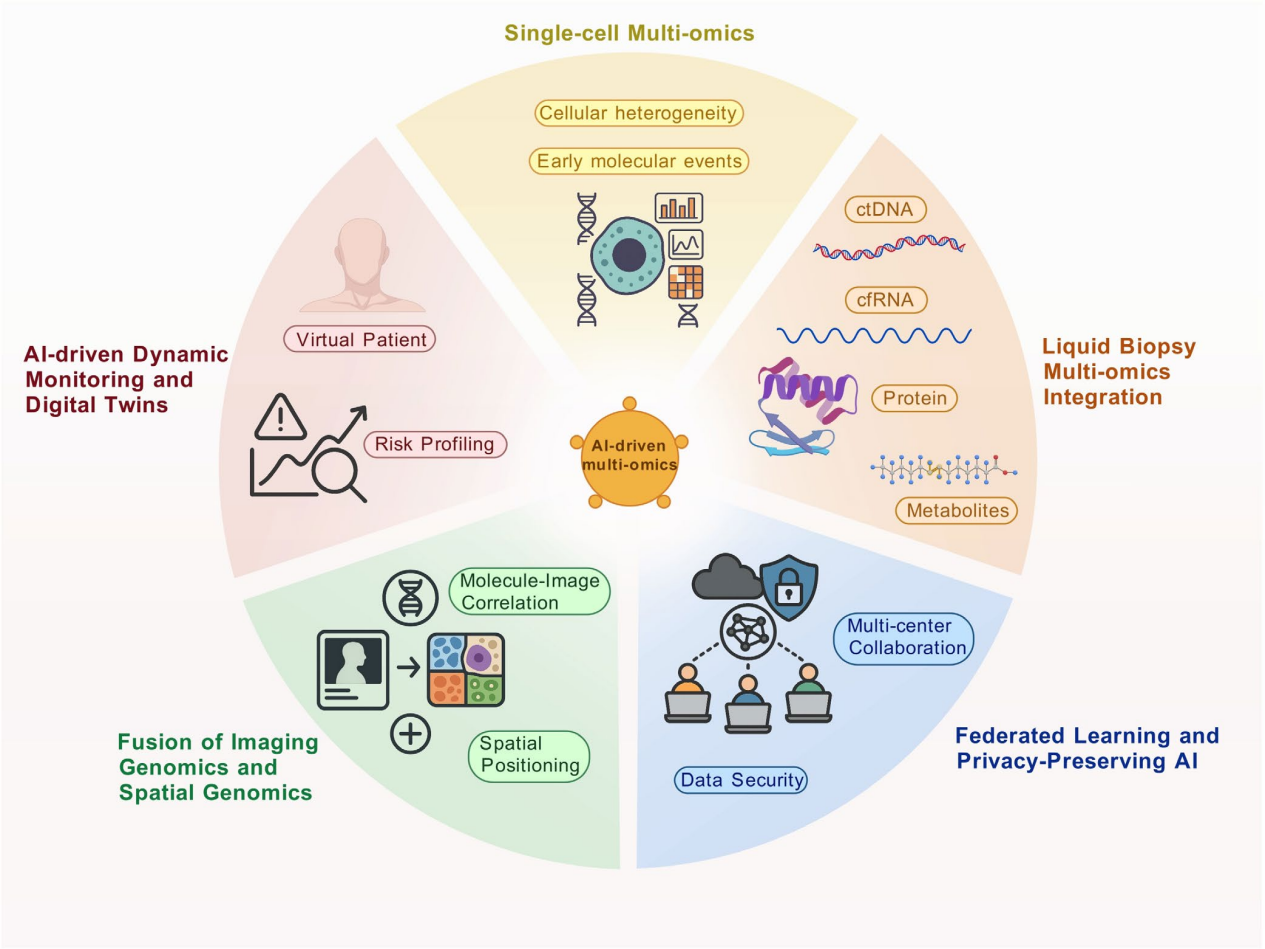
Emerging directions for AI‐driven multi‐omics integration in precision oncology. Generated using BioGDP.com.

Optimisation. For lung cancer, such systems predict individual progression trajectories and personalise screening intervals. Leveraging these advances, Figure 2 illustrates AI‐driven strategies for integrating multi‐omics across precision oncology, mapping future directions for personalised cancer diagnosis, prognosis and therapeutic development. Figure 3 schematically summarises the emerging AI‐driven multi‐omics frameworks for early lung cancer detection, integrating single‐cell and liquid biopsy multi‐omics, imaging–genomics fusion, federated learning, and dynamic patient monitoring.

## 5 Conclusion

This review synthesises recent advances in artificial intelligence (AI) and multi‐omics integration for oncology, focusing on early lung cancer detection and personalised management. We evaluate biomarkers across genomic, epigenomic, transcriptomic, proteomic, metabolomic and microbiomic domains, assessing detection efficacy in liquid biopsies, tissue specimens and non‐invasive samples (e.g., sputum, exhaled breath). AI‐driven machine and deep learning models are shown to extract diagnostic and prognostic insights from high‐dimensional omics data. The analysis examines AI integration into clinical decision support while addressing translational barriers—including data heterogeneity, limited model interpretability, standardisation gaps and ethical considerations. Emerging frontiers such as single‐cell multi‐omics, privacy‐preserving federated learning and radiogenomic fusion represent pivotal approaches for overcoming these challenges and advancing precision oncology.

## Author Contributions

**Fan Bu:** conceptualization; investigation; writing – original draft; methodology; data curation. **Zhi‐Qiang Ling:** conceptualization; methodology; data curation; investigation; validation; supervision; funding acquisition; project administration; writing – review and editing.

## Funding

This work was supported by grants from the 2023 Zhejiang Provincial “Pioneer” and “Pathfinder” R&D Program of the Department of Science and Technology of Zhejiang Province (Grant No. 2023C03055) and the Zhejiang Provincial Health Leading Talent Program (Grant No. Zjwjw2021‐40).

## Ethics Statement

The studies were approved by the Institutional Review Board of Zhejiang Cancer Hospital (Approval No. IRB‐2024‐1277 (IIT)). All data analyses were conducted in strict compliance with relevant guidelines and regulations.

## Consent

All authors read and approved the final manuscript.

## Conflicts of Interest

The authors declare no conflicts of interest.

## Supporting information


**Data S1:** cam471578‐sup‐0001‐GDP‐20250519.pdf.

**Data S2:** cam471578‐sup‐0002‐GDP‐20250521.pdf.

## Data Availability

The datasets used and analysed during the current study are available from the corresponding author on reasonable request.
