# Supplementary material for: Digital Biomarkers for Precision Early Detection of Lung Cancer: Integrating AI‐Driven Multi‐Omics Into Clinical Pathways
Source: Cancer Med. 2026 Feb 5;15(2):e71578. doi: 10.1002/cam4.71578 (PMC12877424; doi:10.1002/cam4.71578)
Supplement: Supplementary file 2 — Data S2: cam471578‐sup‐0002‐GDP‐20250521.pdf. [file CAM4-15-e71578-s001.pdf]

105 Xingang West Road
Haizhu District,Guangzhou,China
BioGDP.com

# Confirmation of Publication and Licensing Rights

BioGDP Account ID: poetefan@outlook.com
Agreement number: GDP20256BE0WQ

To whom this may concern,

This document is to confirm that **Bu Fan** from **Zhejiang Cancer Hospital** has been granted a license to use the BioGDP content, including graphics,templates and other original artwork, appearing in the attached completed graphic pursuant to BioGDP's Academic License Terms.This license permits BioGDP content to be sublicensed for use in **biomarker research** publications.

All rights and ownership of BioGDP content are reserved by BioGDP. All completed graphics must be accompanied by the following citation:“Created with biogdp.com”.

BioGDP content included in the completed graphic is not licensed for any commercial uses beyond publication in a journal.For any commercial use of this figure,users may contact BioGDP Support at gdp-coloring@outlook.com.

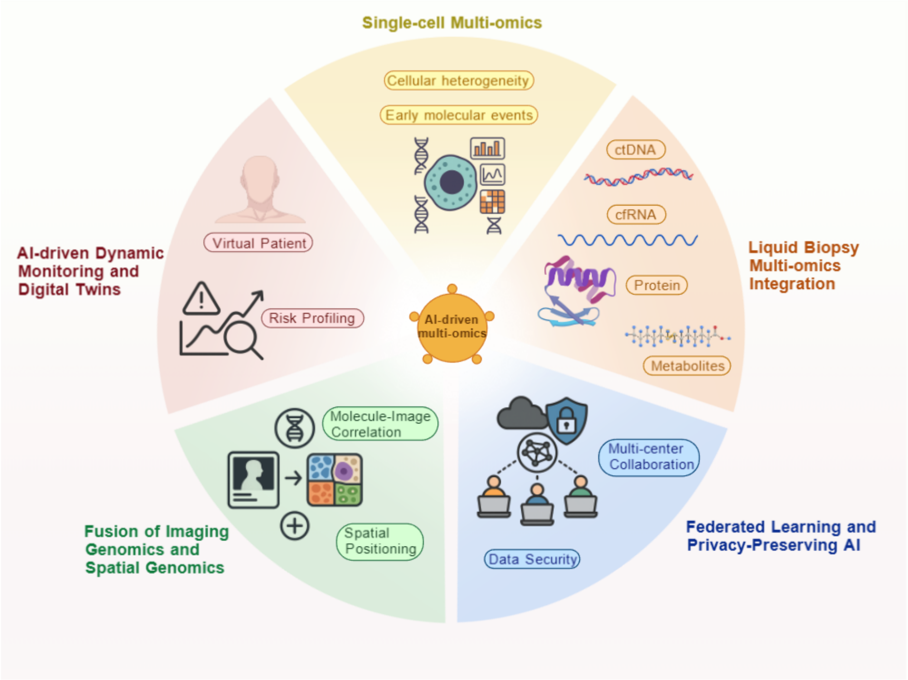


For any questions regarding this document,or other questions about publishing with BioGDP please contact BioGDP Support at gdp-coloring@outlook.com.
